# Untangling Extracellular Proteasome-Osteopontin Circuit Dynamics in Multiple Sclerosis

**DOI:** 10.3390/cells8030262

**Published:** 2019-03-20

**Authors:** Chiara Dianzani, Domizia Vecchio, Nausicaa Clemente, Annalisa Chiocchetti, Filippo Martinelli Boneschi, Daniela Galimberti, Umberto Dianzani, Cristoforo Comi, Michele Mishto, Juliane Liepe

**Affiliations:** 1 Department of Drug Science and Technology, University of Turin, 10126 Torino, Italy; chiara.dianzani@unito.it; 2 Interdisciplinary Research Centre of Autoimmune Diseases (IRCAD), University of Piemonte Orientale, Amedeo Avogadro, 28100 Novara, Italy; domizia.vecchio@gmail.com (D.V.); nausicaa.clemente@med.uniupo.it (N.C.); annalisa.chiocchetti@med.uniupo.it (A.C.); umberto.dianzani@med.uniupo.it (U.D.); cristoforo.comi@med.uniupo.it (C.C.); 3 Department of Biomedical Sciences for Health, University of Milan, 20122 Milan, Italy; filippo.martinelli@unimi.it; 4 MS Research Unit and Department of Neurology, IRCCS Policlinico San Donato, San Donato Milanese, 20097 Milan, Italy; 5 Department of Biomedical, Surgical and Dental Sciences, University of Milan, “Dino Ferrari” Centre, 20100 Milano, Italy; daniela.galimberti@unimi.it; 6 Fondazione IRCCS Cà Granda, Ospedale Maggiore Policlinico, 20100 Milano, Italy; 7 Department of Translational Medicine, Section of Neurology, University of Piemonte Orientale, 28100 Novara, Italy; 8 Centre for Inflammation Biology and Cancer Immunology (CIBCI) & Peter Gorer Department of Immunobiology, King’s College London, SE1 1UL London, UK; 9 Institute for Biochemistry, Charité–Universitätsmedizin Berlin, corporate member of Freie Universität Berlin, Humboldt-Universität zu Berlin, and Berlin Institute of Health, Institut für Biochemie, Germany, 10117 Berlin, Germany; 10 Max-Planck-Institute for Biophysical Chemistry, 37077 Göttingen, Germany

**Keywords:** immunoproteasome, chemotaxis, computational modelling, system biology

## Abstract

The function of proteasomes in extracellular space is still largely unknown. The extracellular proteasome-osteopontin circuit has recently been hypothesized to be part of the inflammatory machinery regulating relapse/remission phase alternation in multiple sclerosis. However, it is still unclear what dynamics there are between the different elements of the circuit, what the role of proteasome isoforms is, and whether these inflammatory circuit dynamics are associated with the clinical severity of multiple sclerosis. To shed light on these aspects of this novel inflammatory circuit, we integrated in vitro proteasome isoform data, cell chemotaxis cell culture data, and clinical data of multiple sclerosis cohorts in a coherent computational inference framework. Thereby, we modeled extracellular osteopontin-proteasome circuit dynamics during relapse/remission alternation in multiple sclerosis. Applying this computational framework to a longitudinal study on single multiple sclerosis patients suggests a complex interaction between extracellular proteasome isoforms and osteopontin with potential clinical implications.

## 1. Introduction

Multiple sclerosis (MS) is a chronic disease of the central nervous system characterized by the presence of inflammation, myelin damage, and axonal degeneration. The causes of MS remain elusive; however, several studies suggest an immune pathogenesis involving a predisposing genetic background in combination with environmental and immune factors [1,2].

Osteopontin (OPN) is not only a component of the bone matrix but also a soluble pleiotropic cytokine. It has been theorized that OPN is a key inflammatory molecule in MS and in other diseases, which is supported by numerous studies [1]. Indeed, OPN concentration increases in bodily fluids during the relapse phase in patients affected by relapsing-remitting multiple sclerosis (RRMS). OPN is abundantly secreted in MS lesions. Further evidence has been provided by studies carried out in experimental autoimmune encephalomyelitis (EAE) mice, which is the main mouse model of MS. In this model, OPN administration results in rapid relapse induction, increased neurological defect levels, and disease progression [3,4].

The secreted form of OPN can be processed by proteases such as matrix metalloproteinases and thrombin [1]. In particular, thrombin cleaves the full length secreted OPN (OPN-FL) into N-terminal (OPN-N) and C-terminal (OPN-C) portions, which exert different biological activities in comparison to OPN-FL. Some of these functions are leucocyte chemotaxis and adhesion, as well as survival and differentiation. It has been hypothesized that this gain of function upon processing could be due to exposure of portions of the OPN protein that are not accessible in the OPN-FL form [1,5]. 

Another protease present in many types of biological fluid is the extracellular 20S proteasome, although this multi-catalytic enzyme is widely studied in its role as an intracellular protease for which it degrades the majority of intracellular proteins. There are different proteasome isoforms with varying catalytic subunits assembled in their barrel-shaped structure. Standard proteasomes are comprised of the β1, β2, and β5 subunits, whereas the immunoproteasome contains the β1i, β2i, and β5i subunits. Immunoproteasomes are normally expressed in cells exposed to an inflammatory milieu or in specific pathological environments, as well as being constitutively expressed in immune cells [6]. These two proteasome isoforms differ in terms of structure, peptide-bond hydrolysis, peptide transport within the proteasome chamber, and transport regulation [7,8]. This results in overall diverse proteolytic dynamics and substrate-specific degradation rates [7,9,10,11,12,13], which are likely responsible for the preferential involvement of the immunoproteasome, for instance, in the regulation of cytokine-mediated inflammation and cell-mediated immunity [14,15]. Immunoproteasome concentration is increased in various cells involved in neurological diseases with inflammatory components, such as Alzheimer’s disease, Huntington’s disease, epilepsy, and MS [16,17,18,19,20,21,22]. Furthermore, immunoproteasomes worsen the phenotype in EAE mice despite the biological mechanisms mediating these effects still being largely unknown [23,24]. 

We recently speculated that the involvement of immunoproteasomes in EAE and MS could be in part due to its regulation of OPN in the extracellular space [25]. What proteasomes do in the extracellular space is still unclear, although we recently identified a reciprocal interaction between extracellular proteasome and OPN, which leads to regulation of cell migration. Proteasome-catalyzed degradation of OPN-FL and OPN-C generates specific peptide fragments present at the C-terminal portion of the protein (from residues 217 to 306). These peptides enhance the migration of different cell types involved in inflammation and angiogenesis, i.e., human umbilical vein endothelial cells (HUVECs), peripheral blood lymphocytes (PBLs), and monocytes (PBMs). In contrast, proteasomal proteolysis significantly hampers the chemotactic activity of OPN-N. In a sort of a feedback loop, OPNs reduce the in vitro secretion of proteasomes by HUVECs but not by PBLs, thereby suggesting cell-specific regulation of this process. This phenomenon seems to be confirmed in vivo in RRMS patients’ sera. Indeed, in the sera of two independent Italian and German cohorts of RRMS patients in relapse or in remission, we measured an increased concentration of OPN during the relapse phase, which significantly correlated inversely with the levels of extracellular proteasomes. In these RRMS cohorts, serum proteasome concentration is larger in patients in remission than in relapse or in comparison to healthy donors. Regarding the mechanism whereby OPNs reduce the secretion of proteasomes in the extracellular space, we detected proteasomes in both extracellular vesicles and vesicle-free supernatant in sera. The proteasome content that is influenced by the RRMS phase seems to be the latter. This suggests a secretion mechanism alternative to extravesicles that has not yet been identified [25].

To untangle the complex implications of the extracellular proteasome-OPN circuit in MS relapse/remission alternation, a pure in vitro/ex vivo experimental strategy would be insufficient, as it could limit the study to an observational level. In this study, we aim for a broader understanding of this phenomenon and the implications that it may have in the clinical history of MS patients. This can be achieved via mathematical modeling coupled to state-of-the-art statistical inference, as has been done in the past to decipher proteasome dynamics [7,26,27,28,29,30].

## 2. Materials and Methods

### 2.1. In Vitro Processing of OPNs by Purified 20S Proteasomes

To begin, 20S proteasomes were purified from T2 and lymphoblastoid cell lines (LCLs), as well as peripheral blood of an RRMS patient in relapse and an age- and gender-matched healthy control, as previously described [31]. The purity of 20S proteasome preparation has been shown elsewhere [9]. LCLs are human B lymphocytes immortalized via Epstein Barr virus transformation that primarily express immunoproteasomes [32]. We pooled LCLs from 5 donors carrying different β1i and β5i subunit polymorphisms to minimize the bias on proteasome activity [19,32,33,34]. The T2 cell line is a human T cell leukemia/B cell line lacking β1i and β5i subunits and thus expressing only standard proteasomes [9]. 

Experiments were carried out using recombinant OPN-FL (OPN_17-314_-6His), OPN-N (OPN_17-168_-6His), and OPN-C (OPN_169-314_-6His), which are here reported as OPNs. The amount of 0.4 µg recombinant OPNs were incubated in kinetics experiments (0–4 h) at 37 °C with 3.5 µg 20S proteasomes purified from either T2 and LCLs (Figure 2A) or from the peripheral blood of an RRMS patient and an age-matched healthy control (Figure S1) in 20 µL Roswell Park Memorial Institute (RPMI) medium. The ratio of OPNs/proteasomes used in the kinetics assays mimicked that observed in serum of healthy and MS donors [25]. OPNs’ degradation was detected and quantified by immunoblotting as previously described [9].

### 2.2. Cell Culture

HUVECs were isolated from human umbilical veins and grown as previously described [25]. The use of primary human cells was approved by the Ethics Committee of the ‘‘Presidio Ospedaliero Martini’’ of Torino and conducted in accordance with the Declaration of Helsinki. Written informed consent was obtained from all donors.

### 2.3. Cell Migration Assay

Cell migration assays were carried out in the Boyden chamber (BD Biosciences) as previously described [25]. Briefly, 2 × 10^3^ HUVECs were plated onto the apical side of 50 µg/mL Matrigel-coated filters in M200 serum-free medium. Ten µg/mL OPN-FL, OPN-C, or OPN-N were placed in the basolateral chamber; 10 ng/mL VEGF-α were used as a reference chemoattractant. 20S proteasome isoforms were pre-incubated with OPN-FL, OPN-C, and OPN-N—with ratio proteasome (ng):OPNs (ng) = 20:1—for 2 h at 37 °C under 5% CO_2_ and subsequently placed in the basolateral chamber. After 3 h, the cells on the bottom of the filter were stained with crystal violet and counted. Data are shown as a percentage of treated cell migration versus the control migration measured for untreated cells. Control migration was (mean ± SD) 87 ± 7.9 for HUVECs (n = 17). The same assay and conditions were used in the migration assays described by Dianzani et al. [25] (and used for developing the computational model) except the cell migration period (20 h instead of 3 h). Furthermore, in the study of Dianzani et al. [25], the PBL migration assay was carried out using 5 × 10^4^ PBLs.

### 2.4. Donor Enrolment and Sample Preparation

RRMS patients and age-, gender-, and ethnicity-matched healthy controls without a history of any neurological or other chronic disease were recruited for the study of Dianzani et al. [25] and are summarized in Table S3. The blood samples of the Italian donor cohort were obtained after informed consent and ethical approval by the University of Piemonte Orientale (Novara; ethical committee approval: CE 18/04) and the University of Milano (ethical committee approval: 2014/020). Diagnosis of MS was made according to the McDonald 2010 criteria [35], and inclusion criteria are described elsewhere [25]. For the longitudinal study, which was not included in the previous study [25], 10 mL of peripheral blood was drawn, and the serum was separated at the time of relapse (2–7 days from its onset) and of remission (after 0.5–8 months from the last relapse; the average period after the relapse is 3 months). The RRMS patients received corticosteroid treatment right after the blood withdrawal during the relapse. Blood of healthy donors was withdrawn at t0 (i.e., at the baseline, 0 months) and 1–5 times within 23 months.

All patients underwent clinical evaluations in relapse and remission, and the expanded disability status scale (EDSS) [36] was filled in at each time-point. To take into consideration the effect of time on disability accumulation, we calculated the multiple sclerosis severity score (MSSS) [37]. The MSSS was based on databases from 11 countries, extracting disease duration (in years) and last EDSS assessment of 9892 patients. The algorithm related a patient’s disability severity according to his/her EDSS to the distribution of disability in others with the same disease duration. In this model, similar relatively high MSSS numbers were assigned to patients who accrued moderate disability over a short period of time or severe disability over a moderate period of time. MSSS was calculated as a decile of the EDSS using the average of the lowest and highest ranks for each possible EDSS at a certain disease duration, which was normalized by dividing by 1 plus the number of available assessments for that year and then multiplied by 10.

To compute the ΔMSSS and the MSSS, we applied the following formulas: ΔMSSS = −(MSSS_remission_ − MSSS_relapse_); MSSS’ = ΔMSSS × MSSS_relapse_. The latter two variables better represent the worsening of symptom severity in the transition from remission to relapse.

### 2.5. Extracellular Proteasome and OPN Quantification in Serum

The concentration of the extracellular proteasomes was measured in serum by ELISA, as previously described [25]. All assays were performed in two biological units and measured in three technical replicates.

### 2.6. Statistical Analysis and Data Availability

Data were tested for normality and homoscedasticity by the Shapiro-Wilk and Kolmogorov-Smirnov tests, and the Levene test, respectively. To identify significant variation in chemotaxis upon different stimuli, we applied the Wilcoxon test for paired samples. Pearson’s correlation coefficients were computed for correlation analyses. A bootstrap test (1000 samples using 100% of the data) was performed to obtain confidence intervals for the correlation coefficient between OPN and 1/extracellular proteasome, as previously described [25]. Descriptive statistics were carried out with SPSS (version 17) and R; a *p*-value < 0.05 was considered statistically significant.

Data are available at Mendeley Data Archive: doi:10.17632/67x4cfgz7p.1.

### 2.7. Mathematical Modeling

The mathematical model consisted of a set of ordinary differential equations that described the time course of extracellular proteasome (*p_i_* and *p_s_* for immuno- and standard- proteasomes), OPNs (*OPN-FL OPN_F_, OPN-N OPN_N_*, and *OPN-C OPN_C_*), and the resulting OPN fragments (*F_sf_, F_sn_,* and *F_sc_* for standard proteasome-generated fragments, *F_if_, F_in_,* and *F_ic_* for immunoproteasome-generated fragments) upon proteasomal degradation. The model parameters were estimated, when possible, from the experimental measurement of serum OPN and proteasome concentrations in RRMS, healthy donors of the Italian cohort [25], and the in vitro degradation of OPNs by standard- and immuno-proteasomes (Figure 2A–B). 

The period of relapse in the MS patients was arbitrarily set to 4 weeks. The model was implemented in R and numerically integrated using LSODA (R package deSolve) [38,39]. All further model analysis was done in R using the packages plot3D [40] and code for parameter inference, and model simulations can be obtained upon request. The details of the model derivation and parameter estimation are as follows:

The Ordinary Differential Equation (ODE) system is defined as:

(1) $$\frac{dp_{i}}{dt}=k_{in}\frac{X_{i}}{1+k_{i}OPN_{tot}}-p_{i}k_{deg}$$

(2) $$\frac{dp_{s}}{dt}=k_{in}\frac{1-X_{i}}{1+k_{i}OPN_{tot}}-p_{s}k_{deg}$$

(3) $$\frac{dOPN_{F}}{dt}=\gamma k_{1}-OPN_{F}\left(\frac{kcut_{F,i}p_{i}}{KM_{F,i}+OPN_{F}}+\frac{kcut_{F,s}p_{s}}{KM_{F,s}+OPN_{F}}\right)-k_{degF}OPN_{F}$$

(4) $$\frac{dOPN_{N}}{dt}=\gamma k_{1}\frac{1-X_{t}}{Xt}-OPN_{N}\left(\frac{kcut_{N,i}p_{i}}{KM_{N,i}+OPN_{N}}+\frac{kcut_{N,s}p_{s}}{KM_{N,s}+OPN_{N}}\right)-k_{degN}OPN_{N}$$

(5) $$\frac{dOPN_{C}}{dt}=\gamma k_{1}\frac{1-X_{t}}{Xt}-OPN_{C}\left(\frac{kcut_{C,i}p_{i}}{KM_{C,i}+OPN_{C}}+\frac{kcut_{C,s}p_{s}}{KM_{C,s}+OPN_{C}}\right)-k_{degC}OPN_{C}$$

(6) $$\frac{dF_{if}}{dt}=p_{i}\frac{kcut_{F,i}OPN_{F}}{KM_{F,i}+OPN_{F}}-F_{if}k_{deg3}$$

(7) $$\frac{dF_{in}}{dt}=p_{i}\frac{kcut_{N,i}OPN_{N}}{KM_{N,i}+OPN_{N}}-F_{in}k_{deg3}$$

(8) $$\frac{dF_{ic}}{dt}=p_{i}\frac{kcut_{C,i}OPN_{C}}{KM_{C,i}+OPN_{C}}-F_{ic}k_{deg3}$$

(9) $$\frac{dF_{sf}}{dt}=p_{s}\frac{kcut_{F,s}OPN_{F}}{KM_{F,s}+OPN_{F}}-F_{sf}k_{deg3}$$

(10) $$\frac{dF_{sn}}{dt}=p_{s}\frac{kcut_{N,s}OPN_{N}}{KM_{N,s}+OPN_{N}}-F_{sn}k_{deg3}$$

(11) $$\frac{dF_{sc}}{dt}=p_{s}\frac{kcut_{C,s}OPN_{C}}{KM_{C,s}+OPN_{C}}-F_{sc}k_{deg3}$$

where *p_s_ and p_i_* are extracellular standard- and immuno-proteasomes, respectively, *OPN_tot_* is the total concentration of OPN-FL, OPN-N, and OPN-C, *OPN_F_* is OPN-FL, *OPN_N_* is OPN-N, *OPN_C_* is OPN-C, X_t_ is the fraction of OPN-FL relative to OPN-C and OPN-N, and X_i_ is the fraction of immunoproteasomes relative to standard proteasomes. OPN-derived fragments generated by immunoproteasome are denoted *F_if_*, *F_in_*, and *F_ic_* for the fragments of OPN-FL, OPN-N, and OPN-C, respectively. OPN-derived fragments generated by standard proteasomes are denoted *F_sf_*, *F_sn_*, and *F_sc_* for the fragments of OPN-FL, OPN-N, and OPN-C, respectively (see also Table S2). The model parameters *k_in_, k_i_, k_deg_, k_1_, k_deg2,_ k_deg3_, KM_F,i_, KM_N,i_, KM_C,i_, KM_F,s_, KM_N,s_, KM_C,s_, kcut_F,i_, kcut_N,i_, kcut_C,i_, kcut_F,s_, kcut_N,s_, kcut_C,s_,* and *k_deg3_* are defined in Table S3. The initial conditions for p_i_, p_s_, OPN_F_, OPN_N_, and OPN_C_ were sampled from the experimental data of Italian RRMS patients in remission [25]. The parameters were inferred from the experimental data [25]. Here, we used three types of data to inform different parameters: (i) remission and relapse measurements of osteopontin and extracellular proteasomes in serum of RRMS patients, (ii) in vitro degradation kinetics of OPNs with purified standard- and immuno-proteasomes, and (iii) chemotaxis assays monitoring migration of HUVECs, lymphocytes, and monocytes in response to unprocessed as well as proteasome-processed OPNs. In particular:

(i) RRMS patient data to estimate transport parameters:

We first focused on determining *k_in_* and *k_deg_*, which describe the rate of proteasome release into the extracellular space and the degradation of extracellular proteasomes, respectively. Both parameters were unknown, but we assumed that during remission, the concentration of extracellular proteasome was constant, i.e., in a steady state. We then had:

$\frac{dp}{dt}=0$ (Equations (1)–(2)), which results in $k_{in}=p_{rem}k_{deg}\left(1+k_{i}OPN_{tot,rem}\right)$, where *p_rem_* is the concentration of extracellular proteasomes during remission, and *OPN_tot,rem_* is the total OPN concentration during remission.

We next determined *k_i_* (rate of proteasome release inhibition by OPNs). Using the steady state experimental data during relapse and remission, respectively, we obtained (Equations (3)–(5)):

$k_{i}=\frac{p_{rel}-p_{rem}}{p_{rem}OPN_{tot,rem}-p_{rel}OPN_{tot,rel}}$, where *p_rel_* is the concentration of extracellular proteasomes during relapse and *OPN_tot,rel_* is the total OPN concentration during relapse. Next, we determined the parameters *k_deg2_* and *k_1_*, which describe OPN degradation and release into the extracellular space, respectively. In steady state during remission, we had: $\frac{dOPN_{F,rem}}{dt}=0$, $\frac{dOPN_{N,rem}}{dt}=0$ and $\frac{dOPN_{C,rem}}{dt}=0$, which resulted in (Equations (3)–(5)):

(12) $$k_{degF}=\frac{k_{1}}{OPN_{F,rem}}-\left(\frac{kcut_{F,i}}{KM_{F,i}+OPN_{F,rem}}+\frac{kcut_{F,s}}{KM_{F,s}+OPN_{F,rem}}\right),$$

(13) $$k_{degN}=\frac{k_{1}}{OPN_{N,rem}}-\left(\frac{kcut_{N,i}}{KM_{N,i}+OPN_{N,rem}}+\frac{kcut_{N,s}}{KM_{N,s}+OPN_{N,rem}}\right)\;\mathrm{and}$$

(14) $$k_{degC}=\frac{k_{1}}{OPN_{C,rem}}-\left(\frac{kcut_{C,i}}{KM_{C,i}+OPN_{C,rem}}+\frac{kcut_{C,s}}{KM_{C,s}+OPN_{C,rem}}\right).$$

During relapse, *k*_1_ is increased by a factor γ, which, based on the experimental data, can be computed as $\gamma =\frac{p_{rel}OPN_{F,rel}}{k_{1}}\left\{\left(\frac{kcut_{F,i}}{KM_{F,i}+OPN_{F,rel}}-\frac{kcut_{F,s}}{KM_{F,s}+OPN_{F,rel}}\right)X_{i}+\frac{k_{degF}}{p_{rel}}\right\}$. To determine the parameters, we drew 1000 samples of OPN and proteasome measurement, obtaining parameter distributions to account for uncertainty.

Thus far, we were able to determine the parameters *k_in_*, *k_degF_, k_degN_, k_degC_, k_i_*, and γ, and only *k_deg,_ k_deg3_* and *k_1_* remained unknown. 

(ii) In vitro OPN degradation kinetics:

In the following, we inferred the parameters related to OPN degradation by standard proteasomes (*KM_F,s_, KM_N,s_, KM_C,s_, kcut_F,s_, kcut_N,s_, kcut_C,s_*) and by immunoproteasomes (*KM_F,i_, KM_N,i_, KM_C,i_, kcut_F,i_, kcut_N,i_, kcut_C,i_*). Since patient-derived data describing OPN degradation by proteasomes in serum could not be experimentally collected, we inferred the related parameters from in vitro digestions of OPNs. The differential equations for the formation of OPN fragments (*F_sf_*, *F_sn_*, and *F_sc_* for standard proteasome, and *F_if_*, *F_in_*, and *F_ic_* for immunoproteasome) in vitro were:

(15) $$\frac{dF_{sf}}{dt}=p_{s}kcut_{F,s}\frac{OPN_{F}}{KM_{F,s}+OPN_{F}}$$

(16) $$\frac{dF_{sn}}{dt}=p_{s}kcut_{N,s}\frac{OPN_{N}}{KM_{N,s}+OPN_{N}}$$

(17) $$\frac{dF_{sc}}{dt}=p_{s}kcut_{C,s}\frac{OPN_{C}}{KM_{C,s}+OPN_{C}}$$

(18) $$\frac{dF_{if}}{dt}=p_{i}kcut_{F,i}\frac{OPN_{F}}{KM_{F,i}+OPN_{F}}$$

(19) $$\frac{dF_{in}}{dt}=p_{i}kcut_{N,i}\frac{OPN_{N}}{KM_{N,i}+OPN_{N}}$$

(20) $$\frac{dF_{ic}}{dt}=p_{i}kcut_{C,i}\frac{OPN_{C}}{KM_{C,i}+OPN_{C}}$$

We used exact Bayesian inference (Metropolis Hastings algorithm implemented in *R*) to infer the posterior parameter distributions based on the experimental data. 

Finally, we obtained all the parameters required for the model simulations (setting *k_deg_* = *k_deg3_* = 2 × 10^−4^ and *k_1_* = 10^−4^). 

(iii) Chemotaxis assays:

We aimed to compute the level of chemotaxis over the progress of the disease phases. We computed the level of chemotaxis as the sum of all chemotactic substances in the system multiplied by their chemotactic index, i.e.:

(21) $$C_{tot}\left(t\right)=\;ci_{OPNF}OPN_{F}\left(t\right)+ci_{OPNN}OPN_{N}\left(t\right)+ci_{OPNC}OPN_{C}\left(t\right)+ci_{Fsf}F_{sf}\left(t\right)+ci_{Fsn}F_{sn}\left(t\right)+ci_{Fsc}F_{sc}\left(t\right)+ci_{Fif}F_{if}\left(t\right)+ci_{Fin}F_{in}\left(t\right)+ci_{Fic}F_{ic}\left(t\right),$$

where *ci* are the chemotactic indices of OPNs and their respective fragments. The values of *ci* were determined based on chemotactic experiments using a given cell type (HUVECs, lymphocytes, and monocytes). In the experimental setup, the OPNs were incubated with either standard- or immuno-proteasomes for 2 h. The reaction mix was then applied in a Boyden chamber experiment, and migrating cells were counted over 20 h. The number of migrated cells relative to control is reported as the overall relative chemotaxis (*crs_OPNF_*, *crs_OPNN_*, *crs_OPNC_* for standard proteasome treatment, and *cri_OPNF_*, *cri_OPNN_*, *cri_OPNC_* for immunoproteasome treatment). Since the chemotactic index describes the relative chemotaxis per nM substance and per time, we solved the following equations for standard proteasomes:

(22) $$crs_{OPNF}=\int_{2}^{22}ci_{OPNF}OPN_{F}dt+\int_{2}^{22}ci_{Fsf}F_{sf}dt,$$

(23) $$crs_{OPNN}=\int_{2}^{22}ci_{OPNN}OPN_{N}dt+\int_{2}^{22}ci_{Fsn}F_{sn}dt,$$

(24) $$crs_{OPNC}=\overset{22}{\underset{2}{\int }}ci_{OPNC}OPN_{C}dt+\overset{22}{\underset{2}{\int }}ci_{Fsc}F_{sc}dt$$

And for immunoproteasome

(25) $$cri_{OPNF}=\int_{2}^{22}ci_{OPNF}OPN_{F}dt+\int_{2}^{22}ci_{Fif}F_{if}dt,$$

(26) $$cri_{OPNN}=\int_{2}^{22}ci_{OPNN}OPN_{N}dt+\int_{2}^{22}ci_{Fin}F_{in}dt,$$

(27) $$cri_{OPNC}=\overset{22}{\underset{2}{\int }}ci_{OPNC}OPN_{C}dt+\overset{22}{\underset{2}{\int }}ci_{Fic}F_{ic}dt$$

In the absence of proteasomes, we obtained the chemotactic indices for OPNs (reported in Table S2). By solving the equations for *ci_FOPNFL_*, *ci_FOPNN_*, and *ci_FOPNC_* as well as numeric integration, we also obtained the remaining three indices. Numeric integration was done with sampled kinetic parameters from the parameter posterior distributions obtained in (ii), which were needed to obtain OPN and OPN-derived fragment concentration over time. This resulted in distributions for the chemotactic indices.

## 3. Results

### 3.1. Modeling the Extracellular Proteasome-OPN Circuit in MS

**Figure 1:**
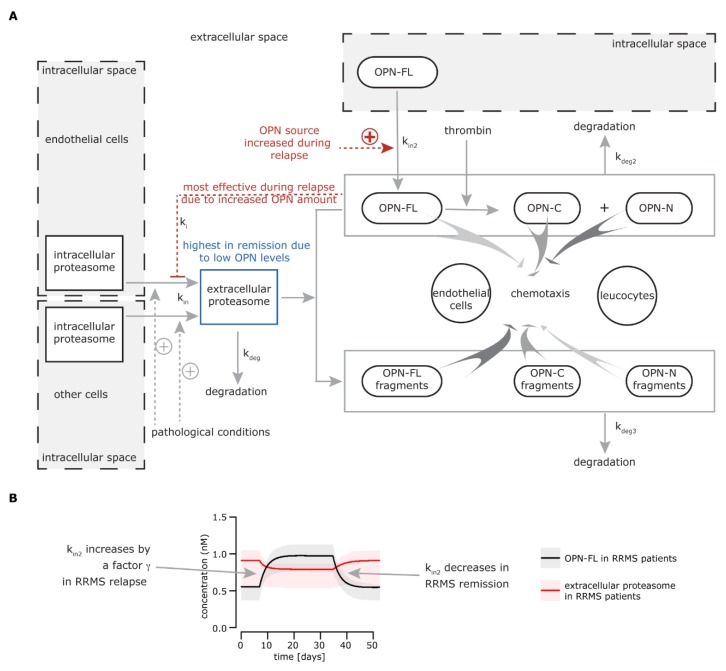
Extracellular osteopontin-(OPN) proteasome circuit model. (**A**) The biological model behind the mathematical model: intracellular proteasomes are released by endothelial cells and other cell types (not identified) into the extracellular space (k_in_), where they can be degraded (k_deg_). This physiological phenomenon could be enhanced in relapsing-remitting multiple sclerosis (RRMS) and other chronic diseases or upon traumatic injury, thereby leading to a chronically larger amount of proteasomes in the extracellular space. The release of proteasomes by endothelial cells is inhibited by OPNs (k_i_) and possibly other unknown factors, whereas other cells are insensitive to OPN-mediated inhibition of proteasome release. k_i_ is prominent during the RRMS relapse when OPN levels are higher, leading to a decrease of proteasome release in the extracellular space. The pathological levels of extracellular proteasomes are restored in the remission when the OPN concentration is diminished. In the extracellular space, full length secreted OPN (OPN-FL) can be fragmented in OPN-C-terminal (OPN-C) and OPN-N-terminal (OPN-N) by thrombin or processed by other proteases. Extracellular proteasomes can cleave OPN-N (k_deg3_), thereby partially compromising its chemotactic activity. The peptide fragments generated by proteasomes via processing OPN-FL and OPN-C (k_deg3_) have an enhanced chemotactic activity compared to the parental proteins, thereby promoting the chemotaxis of different cell types such as endothelial cells, lymphocytes, and monocytes [25]. (**B**) The simulation of OPN-FL and extracellular (standard) proteasome concentrations over time for RRMS patients. The time window is arbitrarily set. The behavior of OPN-FL during relapse is given, and the behavior of extracellular standard proteasomes is a result of the modeled system. Here, we assume that only OPN-FL is present in the extracellular space. The secretion of OPN-FL from cells in the extracellular (k_in2_) increases during (or right before) the relapse in serum of RRMS patients by a γ factor. k_in2_ decreases at the end of the relapse.

Based on the in vitro and ex vivo results previously obtained [25], we could sketch a preliminary model of how the extracellular proteasome-OPN circuit could be involved in the phase alternation in RRMS (Figure 1A). In particular, the release of proteasomes into the extracellular space during traumatic injury or chronic pathological condition was already known [41]. This could explain why the level of serum proteasomes in RRMS patients in remission is significantly higher compared to healthy controls [25], possibly as a consequence of tissue dysfunction and distress. The development of the first relapse in RRMS patients occurs together with (or is caused by) an increase of serum OPN. The high extracellular proteasome content in the patient serum results in enhanced pro-inflammatory stimulation due to enhanced proteasome-mediated OPN processing. It is important to note that 20S proteasomes purified from the peripheral blood of either RRMS patients in relapse or healthy controls degrade all three OPNs in vitro (Figure S1). The pro-inflammatory synergistic effect of extracellular OPNs and proteasomes is rapidly hampered by OPN-mediated inhibition of proteasome release by endothelial (and maybe other) cells in the extracellular space. This is supported by in vitro/ex vivo results showing that OPNs reduce the amount of extracellular proteasomes in HUVEC cell culture, and that there is lower serum proteasome concentration measured in RRMS patients in relapse compared to their remission phase [25]. It is important to note that in the studied RRMS cohort, the blood was withdrawn 2–7 days from the relapse onset. Thus, the hypothetical OPN-mediated inhibition of proteasome release had time to show its effects. 

When negative feedback of OPN was less effective because of the decrease of extracellular OPN concentration, extracellular proteasome levels started to rise up again to a (pathologically) steady state in the remission phase. The mechanism mediating the inhibition of proteasome release in the extracellular space by OPN is still unclear. We identified proteasomes in various extracellular vesicles, although we hypothesized that other (unknown) mechanisms of release may have been involved in the scenario observed in MS patients [25].

### 3.2. Development of a Mathematical Model Describing the Extracellular Proteasome—OPN Circuit during the Recurrence of MS Remission and Relapse Phases

To understand how differing proteolytic preferences of proteasome isoforms can impinge on extracellular proteasome-OPN circuit function in inflammation in the alternation of remission and relapse in RRMS patients, it is mandatory to adopt a computational biology approach. Computational modeling of proteasome dynamics has been successfully applied in the past [7,42]. 

In general, a computational model allows us to test our mechanistic understanding of a biological system through the comparison of model simulations with experimental measurements obtained from the system of interest. However, experimental measurements can only probe a limited number of observables under defined experimental conditions. A computational model allows us to investigate such processes that are hard or impossible to directly measure through experimentation. This is of particular interest when modeling clinical data to describe disease progression in humans, as it is the case for RRMS. Our mechanistic understanding lacks detailed time resolved data of the molecular processes involved in RRMS. The development of novel model and parameter inference approaches describing the information content in experimental data [43,44] provides us with the tools to systematically integrate different data modalities. This allows us to obtain a better mechanistic understanding of the dynamics of the molecular processes during the remission-relapse alteration.

Our in vitro and ex vivo data—presented here and in the previous study [25]—can be summarized conveniently and concisely in a mathematical model that captures the different aspects of the extracellular proteasome-OPN circuit. This model describes the dynamics of extracellular proteasomes, OPN, and proteasome-generated OPN fragments in the context of MS. Proteasomes are released from the cells into the blood and degraded at constant rates, *k_in_* and *k_deg_*. The release of extracellular proteasomes is inhibited by OPN (*k_i_*), which itself is produced and degraded with rates (*k_in2_* and *k_deg2_*). In our modeling scheme, OPN can exist in three forms: OPN-FL, OPN-N, and OPN-C. The different OPNs can additionally be degraded by extracellular proteasomes, resulting in the production of OPN fragments (F_OPNFL_, F_OPNN_, F_OPNC_), which can be further processed and degraded by other peptidases (*k_deg3_*) (Figure 1A; more details are available in the Material and Methods, Table S1, S2).

We first used the model to simulate the time course of extracellular OPNs and proteasomes in RRMS patients (Figure 1B). The initial conditions as well as parameter values were estimated from experimental data obtained from sera of the Italian cohort of healthy donors, RRMS patients in remission, and RRMS patients in relapse [25]. Healthy donors have a lower serum proteasome concentration compared to RRMS patients in remission.

Furthermore, we computed the rate by which OPN inhibited the proteasome release from the difference in extracellular proteasome concentration between relapse and remission in RRMS patients, considering that all three OPN forms had the same potential for inhibition, as previously shown [25]. In our first analysis, we assumed that only OPN-FL was present in blood. We later considered other scenarios because our ex vivo assay could only detect OPN-FL. In healthy donors, the concentration of serum OPNs and proteasomes was lower than that in RRMS patients and was constant over time (data not shown), which led us to assume that they were also constant in RRMS patients during remission. During the onset of the relapse, more OPN was produced (*k_in2_* increased) for two (arbitrary) weeks and remained on its increased level for other two (arbitrary) weeks. After that, the rate of OPN production was set back to its “normal” rate (*k_in2_*), resulting in a decreased serum OPN concentration, i.e., back to its remission level. The factor *γ* by which *k_in2_* increased during relapse was estimated based on extracellular proteasome and OPN levels in RRMS patients during relapse (see Materials and Methods for details).

Thereby, based on the OPN time course, we computed the dynamics of extracellular proteasomes (Figure 1B).

### 3.3. Extracellular 20S Immunoproteasomes Process OPN Molecules Faster and Enhance Their Chemotactic Activity

We initially mentioned existing indirect evidence that hints towards a preferential involvement of immunoproteasomes in MS pathology. To test whether this could be in part due to differential processing of secreted OPNs by immunoproteasomes (as compared to standard proteasomes) in the extracellular space, we measured the in vitro degradation rate of human OPNs carried out by purified standard- and immunoproteasomes. For this set of experiments, we used 20S standard- and immuno-proteasomes. These are the proteasome isoforms that are most likely active in the extracellular space, since they do not require the entire ubiquitin proteasome system to degrade proteins. 20S proteasomes can only efficiently degrade partially disordered proteins because of the reduced size of the proteasome gate [45]. This is the case for OPN, which we have previously shown to be efficiently degraded in vitro by 20S proteasomes [25]. Correspondence between in vitro experiments carried out with purified 20S proteasomes and in cellulo and in vivo experiments has been demonstrated in various studies [12,13,46,47,48,49,50,51,52,53,54,55,56,57].

**Figure 2:**
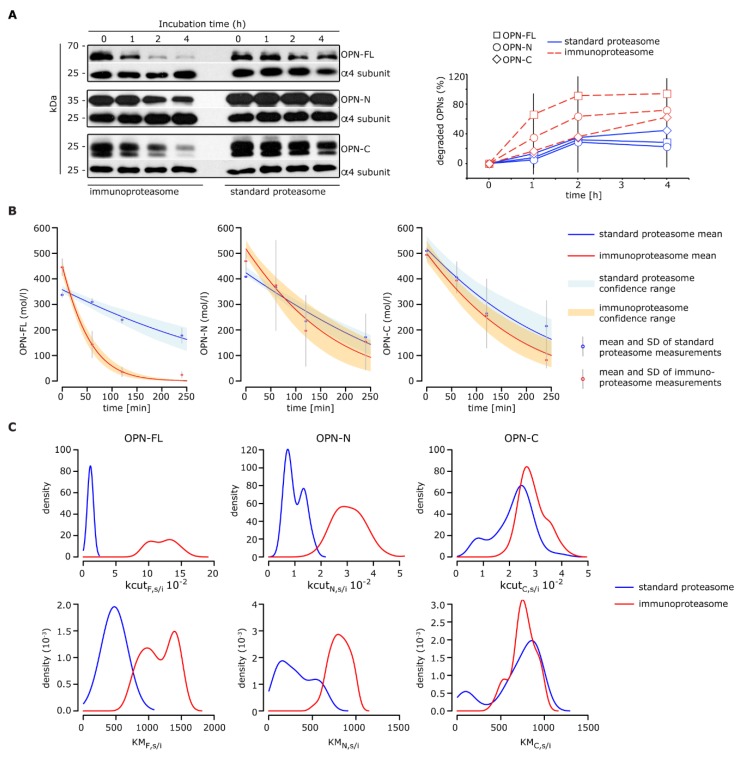
Processing of OPNs by standard- and immuno-proteasomes. (**A**) Representative Western Blot staining of the degradation kinetics of OPNs (OPN-FL, OPN-N, and OPN-C) by 20S standard- and immuno-proteasomes (left panel; the proteasome α4 subunit is used as control marker); the corresponding relative OPN’s degradation is also depicted (right panel; mean and SD of two to three independent experiments measured in duplicate are shown). (**B**–**C**) Inference of kinetic parameters determining the OPNs degradation dynamics by standard- and immuno-proteasomes. Shown are the model fits based on simple Michaelis-Menten kinetics (**B**) and the inferred marginal posterior parameter distributions for *kcut* (maximal reaction velocity) and *KM* (Michaelis-Menten constant) using Bayesian inference via the Metropolis-Hastings algorithm (**C**).

In our experimental set-up, all three OPNs were more rapidly degraded by immuno- rather than standard-proteasomes (Figure 2A). We made use of the in vitro degradation kinetics to estimate the proteasomal processing rates of OPN-FL, OPN-N, and OPN-C in a Bayesian inference scheme (Figure 2B; see Materials and Methods). This allowed us to obtain posterior parameter distributions (rather than point estimates) for OPN processing rates by standard- and immuno-proteasomes (Figure 2C). 

**Figure 3:**
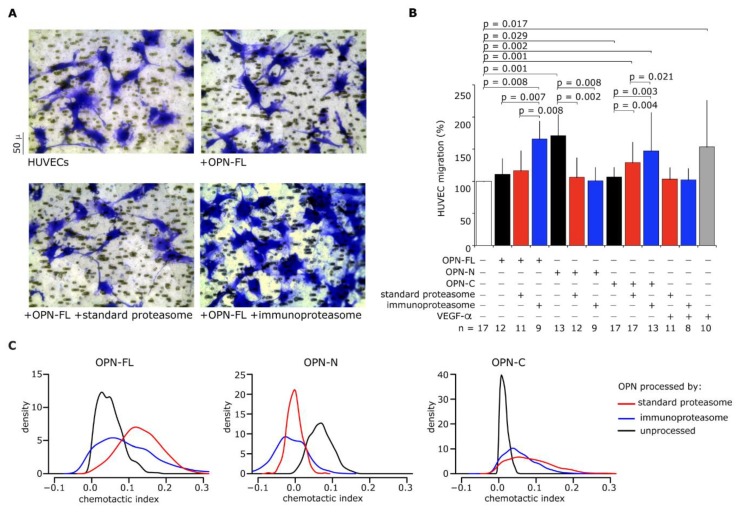
Extracellular immunoproteasomes enhance human umbilical vein endothelial cells (HUVEC) migration more than standard proteasomes through the processing of OPN-FL and OPN-C. (**A**) Images of migrated HUVECs (stained by crystal violet) treated with OPN-FL ± 20S standard- or immuno-proteasomes in a representative Boyden chamber migration assay of 8–17 independent experiments. (**B**) Effect of OPNs (OPN-FL, OPN-N, and OPN-C) ± 20S standard- or immuno-proteasomes, and VEGF-α as positive control, on the HUVEC chemotaxis. Values are the percentage of treated versus untreated cells that migrated after 3 h and are the mean and SD of independent experiments (n = 8–17). Wilcoxon tests for paired samples *p* < 0.05 are shown. (**C**) Chemotactic indices for OPNs and their derived fragments generated by standard- and immuno-proteasomes, respectively. Chemotactic indices are estimated from cell migration experiments with HUVECs.

The different degradation rates of OPNs between standard- and immuno-proteasomes could have had a significant impact on the chemotaxis induced by OPN fragments. Therefore, we measured the HUVECs’ migration upon stimulus with OPNs in the presence or absence of standard- or immuno-proteasomes (Figure 3A). HUVECs are a well-studied model to test chemotactic power of different cytokines, including VEGF-α, which was used here as a positive chemotactic stimulus. In agreement with the in vitro digestion results, when OPN-FL or OPN-C were processed by immunoproteasomes, we observed a significant increase in chemotaxis in comparison to either the unprocessed OPN-FL and OPN-C or the OPN fragments processed by standard proteasomes. In contrast, the chemotactic activity of OPN-N was similarly hampered upon processing by either standard- or immuno-proteasomes (Figure 3B).

By computational modeling, we computed that OPN fragments (F_OPNFL_, F_OPNN_, and F_OPNC_) and OPNs had different chemotactic activities (Figure 3B), which we converted into a chemotactic index expressed as the chemotactic potential of a compound per mol and time (for details, see Methods and Materials). The resulting chemotactic indices for unprocessed OPN-FL and OPN-C were smaller than the chemotactic indices of their proteasome-generated fragments, while the chemotactic index for unprocessed OPN-N was larger than the chemotactic index of its proteasome-derived fragments (Figure 3C). 

Furthermore, immunoproteasomes generated OPN fragments with higher chemotactic index than standard proteasomes (Figure 3C). This suggested a larger generation of chemotactic OPN fragments by immunoproteasomes compared to standard proteasomes, as also indirectly supported by the OPN-FL and OPN-C degradation rates (Figure 2A).

### 3.4. How the Balance between Proteasome Isoforms Can Impinge upon the Chemotaxis in RRMS Phases

We applied the computational model to describe the temporal dynamics of proteasome-generated OPN fragments and the resulting level of chemotaxis in RRMS phases. 

The related parameters were estimated from in vitro and cell culture experiments, as explained above. Because we obtained posterior parameter distributions, we simulated the model 1000 times with parameters sampled from these posterior distributions. We also sampled the initial conditions from the RRMS patient cohort data. This allowed us to estimate expected average behavior of the system and uncertainty, which was propagated through the system, providing confidence ranges for the simulation results.

**Figure 4:**
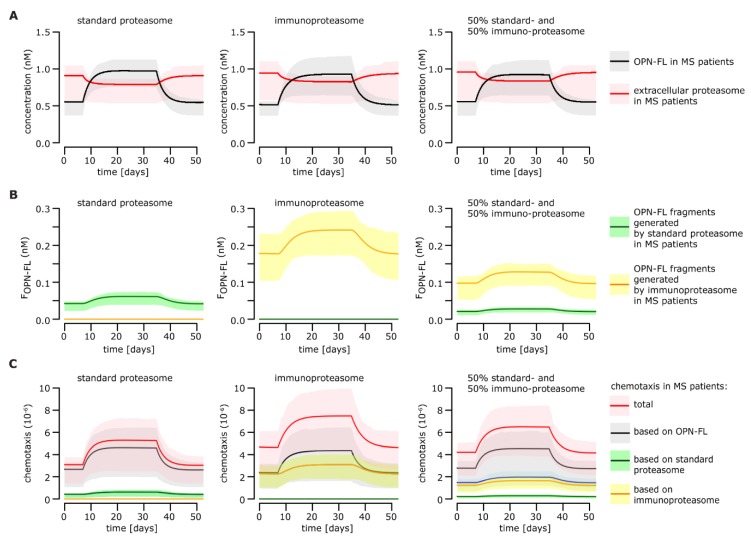
Effect of proteasomes on chemotaxis during RRMS relapse and remission. All simulations are done for standard proteasomes only (left column), immunoproteasomes only (middle column), and 50% standard- and immuno-proteasomes (right column). (**A**) Simulation of OPN-FL and extracellular proteasome concentrations over time in MS patient serum. The behavior of OPN-FL concentration during relapse is given, and the behavior of extracellular proteasome concentration is a result of the modeled system. Here, we assume that only OPN-FL is present in the extracellular space. (**B**) Simulation of the time course of proteasome-generated OPN fragments in MS patient serum. (**C**) Level of chemotaxis over the course of the disease in MS patients. The chemotaxis is computed based on the cell migration experiments performed with HUVECs (Figure 3). Additionally, to the overall expected chemotaxis levels, the chemotaxis based only on OPN-FL, only on standard proteasomes, and only on immunoproteasomes is shown, respectively.

By applying the computational model developed thus far, we could describe the variation of serum OPN and proteasome concentration during the relapse and the remission before and after the relapse in an archetypical RRMS patient. Because we showed that the OPN degradation rate could depend on the proteasome isoform, in the model simulations, we distinguished between three scenarios where (i) only standard proteasomes, (ii) only immunoproteasomes, and (iii) 50% standard- and immuno-proteasomes were present in the serum (Figure 4A). Since OPNs were faster degraded by immuno- rather than standard-proteasomes (Figure 2A), the model simulations suggested that more OPN fragments would be generated if serum proteasomes were only immunoproteasomes (Figure 4B). 

Since the OPN fragments (F_OPNFL_, F_OPNN_, and F_OPNC_) and the OPNs had different chemotactic activities, the overall chemotaxis was expected to be altered depending on the proportion of standard- and immuno-proteasomes. We used the estimated chemotactic indices to simulate the chemotactic activity over time during the remission and relapse of RRMS patients (Figure 3C). 

According to our model simulations, the chemotaxis promoted by OPNs quantitatively prevailed over that promoted by OPN fragments (Figure 4C). The chemotaxis was, however, affected by the proteasome isoform ratio. Immunoproteasomes triggered stronger OPN-mediated chemotaxis than standard proteasomes, which benefited from a stronger impact of OPN-derived fragments on the overall chemotaxis (Figure 4C).

### 3.5. How the Processing of OPN-FL by Thrombin Can Affect the Extracellular OPN-Proteasome Circuit

**Figure 5:**
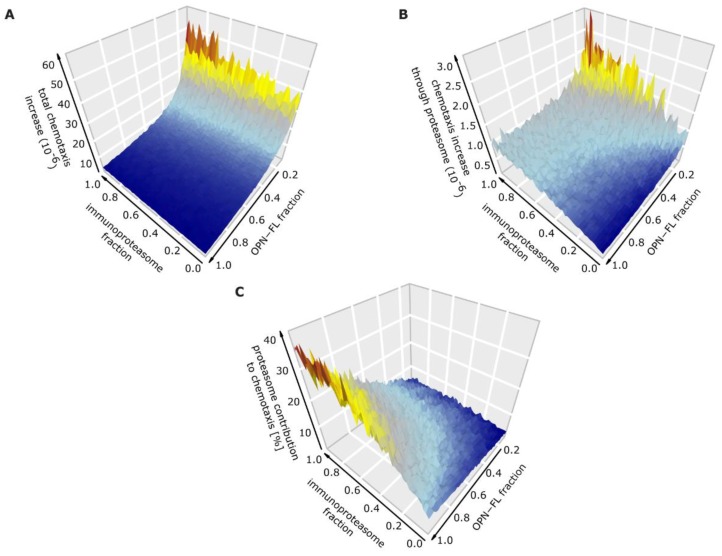
Effect of proteasome isoforms and thrombin on chemotaxis in RRMS patients. (**A**) Overall expected intensification in chemotaxis from RRMS remission to relapse dependant on the ratio of standard- and immuno-proteasomes, as well as on the ratio of OPN-FL compared to OPN-N and OPN-C. (**B**) Expected increase in chemotaxis from RRMS remission to relapse based only on proteasome-generated OPN fragments. (**C**) Relative contribution of proteasome isoforms to the overall expected level of chemotaxis.

Although relevant to the inflammatory behavior, we did not have any information concerning the OPN-FL/OPN-N/OPN-C ratio in the sera of healthy donors and MS patients. This ratio would depend on a few proteases including proteasomes, since the latter degrades OPN-N and OPN-C with different rates (Figure 2A). Therefore, we computed the chemotaxis for various concentrations of OPNs and proportions of standard- versus immuno-proteasomes. In particular, we simulated the increase in chemotaxis from the remission to the relapse in the archetypal RRMS patient to describe how the variation of the ratios of OPNs and proteasome isoforms in blood could impinge upon the cell migration dynamics of the relapse onset (Figure 5). The model simulations suggested that the higher the thrombin activity in blood was (less OPN-FL and more OPN-N and OPN-C), the larger the difference in chemotaxis between relapse and remission was (Figure 5A).

Furthermore, the more prevalent immunoproteasomes are in the blood (compared to standard proteasome), the higher the chemotaxis difference between relapse and remission is (Figure 5B).

Our model simulations also suggested that extracellular proteasomes had the strongest effect on chemotaxis when less thrombin was present in the blood (Figure 5C). This latter outcome indicated that, in the absence of thrombin, the balance of proteasome isoforms could significantly alter the level of cell chemotaxis and therefore the regulation of the inflammatory response in RRMS.

### 3.6. Extension of the Model to Immune Cells Infiltrating the Central Nervous System (CNS) of MS Patients

**Figure 6:**
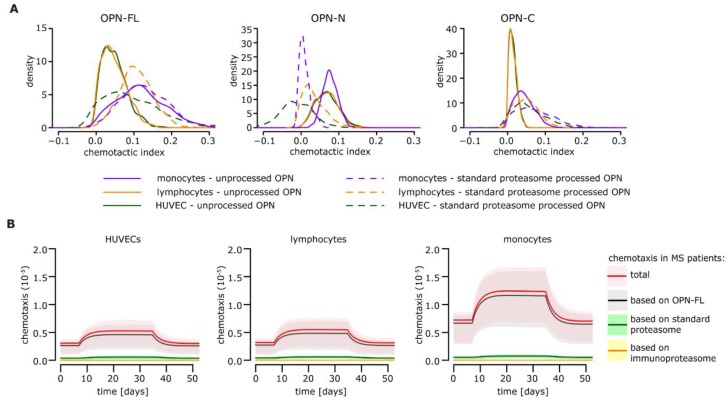
Extracellular OPN and proteasome dynamics and related chemotaxis for HUVECs, lymphocytes, and monocytes. (**A**) Chemotactic indices of OPNs (OPN-FL, OPN-N, and OPN-C) as well as their derived fragments generated by standard proteasomes for HUVECs, lymphocytes, and monocytes. (**B**) Chemotaxis levels over the course of the disease for MS patients based on cell migration experiments [25] performed with HUVECs (left), lymphocytes (middle), or monocytes (right). Simulations are based assuming that only standard proteasomes (absence of immunoproteasome) and only OPN-FL (absence of thrombin) are present in the system.

HUVECs are an established model for cell migration. However, in the pathophysiology of MS, the cells that infiltrate the central nervous system (CNS) and are involved in brain damage are immune cells. Among them, lymphocytes and monocytes are the most studied. They cross the blood-brain-barrier, also attracted by OPN, and in the CNS, they promote inflammation and damage of the parenchyma [1]. We therefore verified whether these cells showed altered chemotactic behavior in response to OPNs and proteasomes, and what could happen in the RRMS alternation phase. We repeated these analyses by studying the migratory behavior of lymphocytes and monocytes and subsequently estimating their chemotactic indices (Figure 6A). The model simulations were generated based on the lymphocyte and monocyte migration ex vivo assays performed with OPNs and standard proteasomes [25]. Overall, we found that the migration behavior in response to OPNs of lymphocytes and monocytes was comparable to that of HUVECs, although monocytes showed higher levels of chemotaxis in the absence of thrombin (Figure 6B) due to higher chemotactic indices resulting from OPN-Fl (Figure 6A). This consistency between HUVECs and immune cells hinted toward a similar impact of standard- and immune-proteasomes on the extracellular proteasome-OPN circuit. Therefore, we could preliminarily extend the outcomes of the mathematical modeling of the cell migration in the presence of standard- and immune-proteasomes—which were based on in vitro results obtained with HUVECs—to the immune cells infiltrating the MS brain.

### 3.7. How the Extracellular OPN-Proteasome Circuit Could Impinge upon the Severity of the Relapse in RRMS Patients

The proposed model of the extracellular proteasome-OPN circuit in MS (Figure 1A) was based on the correlation of serum OPNs and proteasomes measured in Italian cohorts of RRMS patients, whose blood was withdrawn either in the relapse or in the remission phase [25]. The fact that the samples from the relapse and remission phases were not from the same patient could have caused bias. Therefore, we measured the concentrations of OPNs and proteasomes in the sera of a new Italian cohort of RRMS patients (n = 21), which was withdrawn during the relapse and different remission periods (Table S3). The sera during the relapse was collected before steroid treatment to avoid interference by the anti-inflammatory drug on our measurements; between one and four samples from each patient were collected during the remission phase (i.e., 1–8 months after the relapse; average period of three months). Five RRMS patients were excluded from the final analysis because of an incomplete set of values.

**Figure 7:**
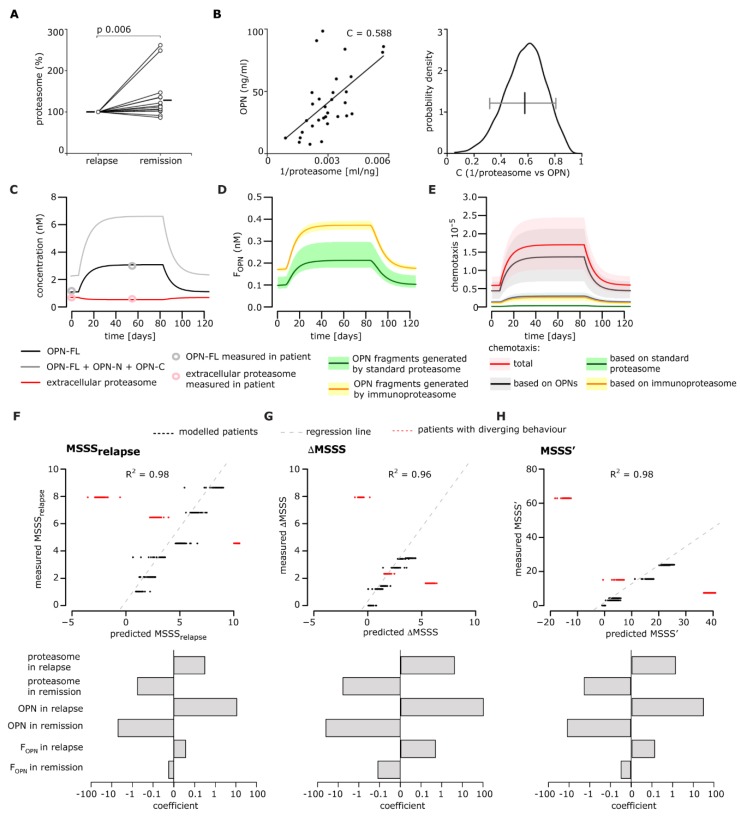
Correlation between relapse severity and serum OPN-proteasome dynamics in an RRMS patient follow-up cohort. (**A**) Variation of proteasome concentration between relapse and remission in the sera of Italian RRMS patients (n = 16). In case of repeated withdrawals during the remission phase (one to three withdrawals for each donor carried out between one and eight months), we calculated the average serum proteasome and OPN levels of the repeated withdrawals and used it for the Wilcoxon test for paired samples (*p* < 0.05 is shown). The means are depicted by a solid line. We obtained a *p* = 0.010 when we excluded the two outliers with a relative proteasome concentration in remission above 200% (n = 14). (**B**) Correlation between proteasome and OPN levels in sera withdrawn during the relapse and remission phases (Pearson’s test, *p* < 0.001; C value is shown in the chart). We included all measurements in the RRMS patients (n = 21) regardless of the MS phase. Therefore, for each patient, one to five values are reported, which correspond to the number of serum samples in which we successfully measured OPN and proteasome concentrations. A Bootstrap test using 90% of the data showed a significant correlation coefficient between 0.3 and 0.8 (right panel). (**C**–**E**) Example model simulation for a single RRMS patient [MS severity score (MSSS) = 23.8]. (**C**) Simulation of OPN-FL, total OPNs, and extracellular proteasome concentrations over time for MS patients. For each single patient simulation, we assumed a ratio of OPN-FL/(OPN-N + OPN-C) and standard-/immuno-proteasomes equal to 0.5 in the extracellular space. (**D**) Time course of standard- and immuno-proteasome-generated OPN fragments in the representative RRMS patient over time. (**E**) Level of chemotaxis over the course of the disease in the representative RRMS patient. The chemotaxis was computed based on cell migration experiments performed with HUVECs (Figure 3). In addition to the overall expected chemotaxis levels, the chemotaxis based only on OPNs, only on standard proteasomes, and only on immunoproteasomes is shown. (**F**–**H**) Performance evaluation of generalized linear models to detect a relationship between the concentration of the extracellular OPN-proteasome circuit components (as in Figure 1) and the relapse severity variables, expressed as MSSS (**F**), ΔMSSS (**G**), and MSSSS’ (**H**). The model was generated using the six RRMS patients (group A) who had the expected variations of serum OPNs and proteasomes from remission to relapse (black dots). The predicted and recorded MSSS, ΔMSSS, and MSSSS’ of the further three RRMS patients (group B) who did not have the expected variations of serum OPNs and proteasomes from remission to relapse are depicted in red dots (**F**–**H** upper panels). In the lower panels of (**F**–**H**) are the coefficients of the generalized linear models to detect relationships between the concentrations of extracellular OPN-proteasome circuit components and MSSS (**F**), ΔMSSS (**G**), and MSSSS’ (**H**). Only significant components are shown.

In the remaining 16 RRMS patients, the serum proteasome concentration was significantly larger in remission than relapse phase in a paired-sample analysis (Figure 7A), agreeing with our previous study [25]. Furthermore, this allowed for confirmation of the (non-linear) inverse correlation between serum OPN and proteasome levels (Figure 7B).

One of the ultimate goals of this study was to verify whether there was a correlation between the extracellular OPN-proteasome circuit and the RRMS patient’s disability. To do that, we considered the EDSS of the RRMS patients enrolled in the prospective study. For nine out of 16 RRMS patients, we could compute the MSSS, a quantitative measure of a patient’s disability corrected for disease duration during relapse and remission, as well as the ΔMSSS and the MSSS’, which represent the MSSS variation from remission to relapse.

In an initial analysis, no correlation between the MSSS, ΔMSSS, or MSSS’ and either the OPN or proteasome concentration in serum was detected. Therefore, we applied our computational model to simulate each patient separately and test whether the dynamics of each extracellular OPN-proteasome circuit component might be correlated with the disability’s increase from remission to relapse. Since the ratio of standard- versus immuno-proteasomes and between OPNs is not known, we simulated each patient assuming 50% standard- and 50% immuno-proteasome, as well as 50% OPN-FL compared to OPN-N and OPN-C. The simulation of the extracellular proteasome-OPN concentrations, the predicted dynamics of proteasome-generated OPN fragments, and the resulting chemotaxis of a representative patient are shown in Figure 7C–E. Among the nine patients, six showed the expected increase of OPN and the expected decrease of proteasome concentrations from remission to relapse (group A), whereas three patients showed diverging behavior (group B; see Figure 7F–H). After simulating each patient based on their OPN and proteasome levels in remission and relapse, we tested whether the model simulations in relapse and remission could be correlated with either the MSSS, ΔMSSS (data not shown), or MSSS’ (Figure S2) of each patient in group A. No correlation could be detected for the single pathway components.

We then tested whether we could predict the MSSS, ΔMSSS, and MSSS’ of each patient based on a combination of the model components by fitting a generalized linear model with 10-fold cross-validation. To this end, we generated models of various complexity in terms of input variables, ranging from including only OPNs during relapse and remission to including all model variables. We evaluated model performance via computation of the R^2^ value. We found that a linear model based on the concentration of OPNs, proteasomes, and proteasome-generated OPN fragments during remission and relapse showed the best performance to predict MSSS, ΔMSSS, and MSSS’ (Figure 7F–H).

By including more model components such as chemotaxis levels or distinguishing between standard- and immuno-proteasome levels, we did not observe any significant performance improvement (data not shown). Linear models not considering proteasomes and proteasome-generated OPN fragments had very poor model performances predicting MSSS, ΔMSSS, and MSSS’ (R^2^ = 0.09, 0.05, 0.14, respectively). Linear models not considering OPNs and the resulting chemotaxis of those chemokines, i.e., including only proteasomes and proteasome-generated OPN fragments, had better model performances predicting MSSS, ΔMSSS, and MSSS’ (R^2^ = 0.67, 0.66, 0.74, respectively).

This outcome indicated that the concentration of extracellular proteasomes, OPNs, and their derived fragments impinged upon the worsening of symptoms during the relapse of RRMS patients in group A. By applying the same linear model to simulations based on the three group B patients, we observed a strong discrepancy between their predicted MSSS’ values and the measured MSSS, ΔMSSS, and the MSSS’ (Figure 7F–H, red dots). This suggested that the linear model could only predict the MSSS, ΔMSSS, and MSSS’ of RRMS patients who had a variation of serum OPN and proteasome concentration from remission to relapse. This is represented in Figure 1B, i.e., an increase of OPNs and a decrease of extracellular proteasomes from remission to relapse.

It is important to note that the absolute values of the estimated coefficients of the linear model were highest for the OPN concentration, and all remission coefficients were positive, while all relapse coefficients were negative, indicating the importance of the change between remission and relapse (Figure 7F–H).

## 4. Discussion

Despite the extensive presence of proteasomes in extracellular space, its function there is far from being completely understood. Recently, we showed that extracellular proteasomes can regulate some aspects of the immune response by interacting with OPN [25]. Using computational modeling, we better defined the interplay between serum OPNs and proteasomes in relapse/remission alternation in RRMS patients and how this interaction could affect chemotaxis. By applying our model, we simulated the correlation between the serum OPN-proteasome circuit and the severity of the relapse in RRMS patients followed over time in a prospective study. These computational simulations depict a complex picture in which the final effect of modulation on the extracellular OPN-proteasome circuit depends on several variables. Only some of these variables were experimentally tested in this study. In our opinion, the model could streamline its efficacy once some biological aspects of the pathological mechanism are experimentally defined. In particular, the model was based on the concentration of proteasomes and OPNs in serum. This bodily fluid may represent only part of the concentration of OPNs and proteasomes—and thus the OPN-proteasome ratio—in the extracellular space of the CNS focal lesions. We speculate that the extracellular OPN-proteasome inflammatory circuit plays a significant role in the regulation of RRMS inflammation in the perivascular cuffing, the cerebrospinal fluid (CSF), and the CNS, where the concentrations of the extracellular OPN-proteasome circuit components have only been partially measured [1,58]. In these regions, the extracellular OPN-proteasome circuit dynamics could be significantly different to what was measured in the serum and could be simulated by our computational model. A second element to bear in mind is that the model does not account for some other factors that can influence the extracellular OPN-proteasome circuit functionality, such as the presence of autoantibodies against proteasomes and OPNs. They can affect the amount and activity of free OPNs and proteasomes and have been correlated to MS and EAE dynamics [59,60,61]. Our ELISA detection method cannot directly address this matter. These latter factors might play a role in the differences between the RRMS patient groups A and B in our longitudinal study. A third variable to be evaluated is the OPNs’ and proteasome isoforms’ ratios in the serum and CNS. Indeed, the computational model simulates the impact that variations of the relative concentration of OPN-FL versus OPN-N as well as standard- versus immuno-proteasomes can have on chemotaxis. However, the direct experimental measurement of the concentration of these specific molecules in the serum and CNS focal lesions could improve the computational model and its applications. It is important to note that we previously described an increased expression of the immunoproteasome subunit β1i in MS plaques as compared to non-MS specimens [19]. Those results were obtained using immunohistochemistry. They showed a clear expression of the immunoproteasome subunit β1i in oligodendrocytes, macrophages, and microglial cells in MS plaques. They also hinted towards a signal specific to the subunit β1i in the extracellular space of MS plaques, which was not observed in non-MS specimens [19].

Only upon including these factors in the computational model could we simulate the actual effect of a potential therapy inhibiting extracellular proteasome activity in RRMS patients.

## Supplementary Materials

The following are available online at https://www.mdpi.com/2073-4409/8/3/262/s1, Figure S1: Proteasome derived from serum of either RRMS patient of healthy control can degrade OPNs; Figure S2: The MSSS’ cannot be predicted based on single components of the extracellular OPN-proteasome circuit; Table S1: Mathematical model variables; Table S2: Mathematical model parameters; Table S3: Characteristics of healthy and RRMS donors enrolled in the prospective study.
