# Supplementary material for: Untangling Extracellular Proteasome-Osteopontin Circuit Dynamics in Multiple Sclerosis
Source: Cells. 2019 Mar 20;8(3):262. doi: 10.3390/cells8030262 (PMC6468732; doi:10.3390/cells8030262)
Supplement: Supplementary file 1 [file cells-08-00262-s001.pdf]

Untangling extracellular proteasome-osteopontin circuit dynamics in multiple sclerosis

Dianzani *et al*.

List of supplementary material

Table S1	Mathematical model variables
Table S2	Mathematical model parameters
Table S3	Characteristics of healthy and RRMS donors enrolled in the prospective study
Figure S1	Proteasomes derived from serum of either RRMS patient or healthy controls can degrade OPNs
Figure S2	The MSSS' cannot be predicted based on single components of the extracellular OPN-proteasome circuit

variable	description
p_s	Standard proteasome
p_i	immunoproteasome
OPN_F	OPN-FL
OPN_N	OPN-N
OPN_C	OPN-C
F_{sf}	OPN-FL-derived fragments produced by standard proteasome
F_{sn}	OPN-N-derived fragments produced by standard proteasome
F_{sc}	OPN-C-derived fragments produced by standard proteasome
F_{if}	OPN-FL-derived fragments produced by immunoproteasome
F_{in}	OPN-N-derived fragments produced by immunoproteasome
F_{ic}	OPN-C-derived-derived fragments produced by immunoproteasome

Table S1. Mathematical model variables.

parameters	description
transport dynamics	
k_{in}	rate of proteasome release into blood vessel
k_{deg}	rate of proteasome degradation
k_1	rate of OPN release into blood vessel
k_{degF}	rate of OPN-FL degradation
k_{degN}	rate of OPN-N degradation
k_{degC}	rate of OPN-C degradation
k_i	rate of inhibition of proteasome release by OPN
X_t	proportion of initial OPN-FL compared to OPN-N and OPN-C
k_{deg3}	rate of degradation of OPNs-derived fragments
proteasomal OPN degradation	
$kcut_{F,s}$ $kcut_{F,i}$	v_{max} of OPN-FL degradation by standard- and immuno-proteasomes, respectively
$Kcut_{N,s}$ $kcut_{N,i}$	v_{max} of OPN-N degradation by standard- and immuno-proteasomes, respectively
$Kcut_{C,s}$ $kcut_{C,i}$	v_{max} of OPN-C degradation by standard- and immuno-proteasomes, respectively
$KM_{F,s}$ $KM_{F,i}$	Michaelis-Menten constant for OPN-FL degradation by standard- and immuno-proteasomes, respectively
$KM_{N,s}$ $KM_{N,i}$	Michaelis-Menten constant for OPN-N degradation by standard- and immune-proteasomes, respectively
$KM_{C,s}$ $KM_{C,i}$	Michaelis-Menten constant for OPN-C degradation by standard- and immuno-proteasomes, respectively
chemotaxis	
ci_{OPNF}	chemotactic index of OPN_F
ci_{OPNN}	chemotactic index of OPN_N
ci_{OPNC}	chemotactic index of OPN_C
ci_{Fsf}	chemotactic index of F_{sf}
ci_{Fsn}	chemotactic index of F_{sn}
ci_{Fsc}	chemotactic index of F_{sc}
ci_{Fif}	chemotactic index of F_{if}
ci_{Fin}	chemotactic index of F_{in}
ci_{Fic}	chemotactic index of F_{ic}

Table S2. Mathematical model parameters.

	Healthy Controls	RRMS
Age (y)	31.8±6.7	36.9±9.1
Count (n)	12	21
Gender (M/F)	3/9	7/14
Disease onset (y)	-	30.3±8.7
Disease duration (y)	-	6.9±6.2

Table S3. Characteristics of RRMS donors enrolled in the prospective study. RRMS patients and healthy donors enrolled in the study from whom we have successfully measured the concentration of proteasome and OPN in at least one serum sample.

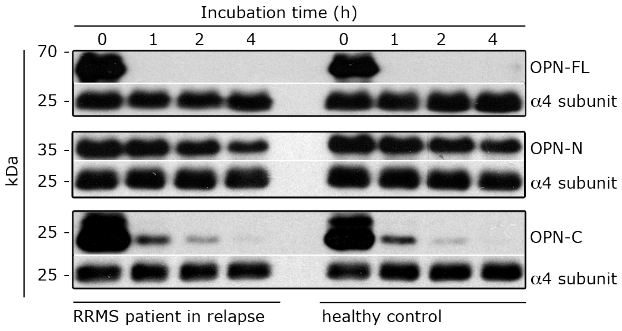

Figure S1. Proteasomes derived from serum of either RRMS patient or healthy controls can degrade OPNs. Degradation kinetics of OPNs (OPN-FL, OPN-N and OPN-C) by 20S proteasomes purified from whole blood of either one RRMS patient or an age-matched healthy control are shown by representative Western Blot assay (upper panel; the proteasome α4 subunit is used as control marker).

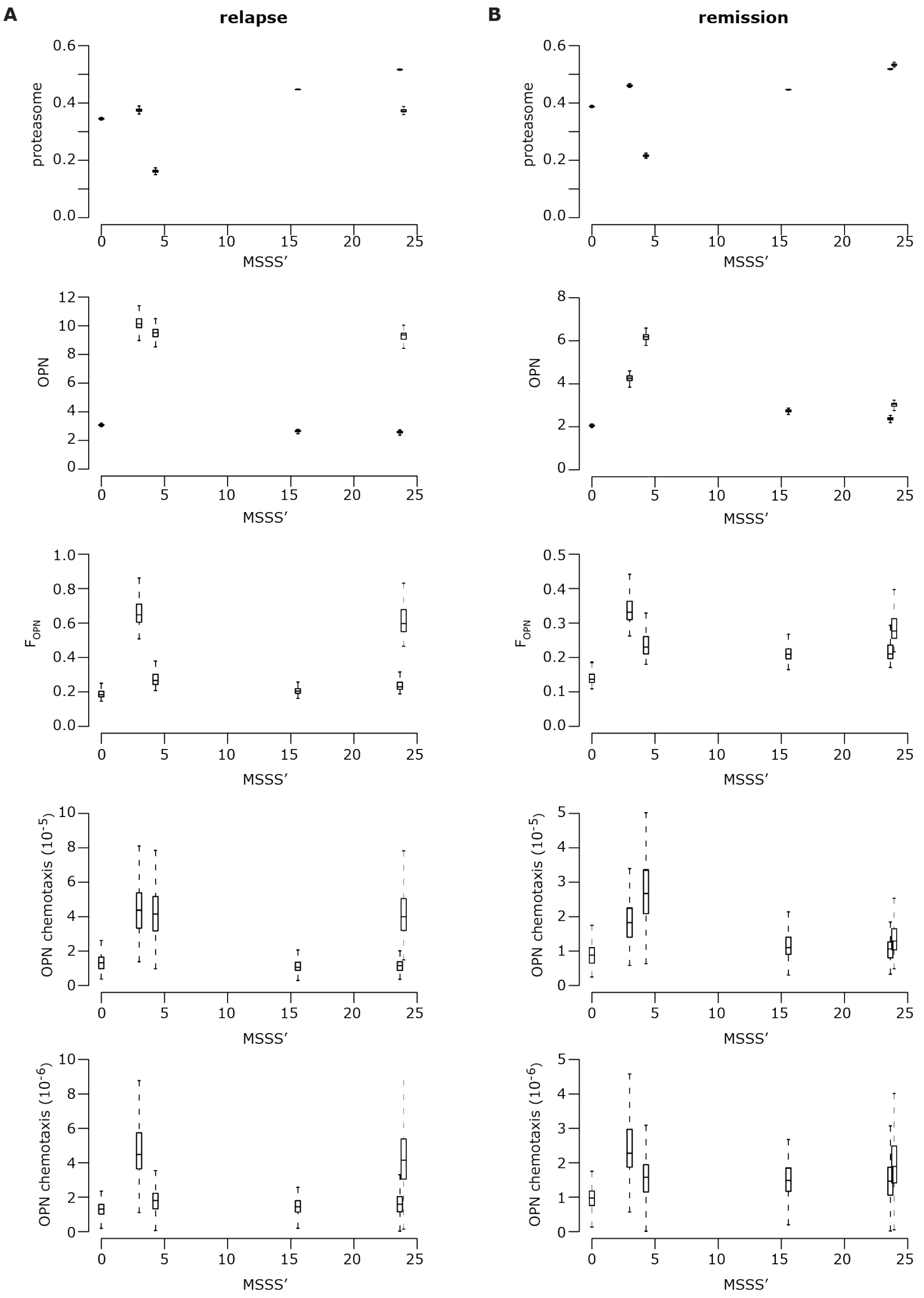

Figure S2. The MSSS' cannot be predicted based on single components of the extracellular OPN-proteasome circuit. Simulated pathway components based on single patient data (the six patients of group A) in relapse (**A**) and remission (**B**) plotted against the patients' MSSS' values. MSSS' is a marker of the relapse clinical intensity and is defined as the MSSS variation from remission to relapse multiplied by the MSSS in relapse ($\Delta MSSS*MSSS_{relapse}$). No correlations between single components and MSSS' have been detected. Pathway components are plotted as boxplots, indicating the median, 25% and 75% quantiles as the box, and the 5% and 95% quantiles as dashed lines.
